# Preventing Axonal Sodium Overload or Mitochondrial Calcium Uptake Protects Axonal Mitochondria from Oxidative Stress-Induced Alterations

**DOI:** 10.1155/2022/6125711

**Published:** 2022-05-24

**Authors:** Rebecca Ulshöfer, Helena Bros, Anja Erika Hauser, Raluca Aura Niesner, Friedemann Paul, Bimala Malla, Carmen Infante-Duarte

**Affiliations:** ^1^Experimental and Clinical Research Center (ECRC), A Cooperation between Charité-Universitätsmedizin Berlin and Max-Delbrück-Center for Molecular Medicine, 13125 Berlin, Germany; ^2^Charité-Universitätsmedizin Berlin, Corporate Member of Freie Universität Berlin, Humboldt Universität zu Berlin and Berlin Institute of Health, 10117 Berlin, Germany; ^3^Max-Delbrück-Center for Molecular Medicine (MDC) in the Helmholtz Association, 13125 Berlin, Germany; ^4^Charité-Universitätsmedizin Berlin, Institute for Medical Immunology, Augustenburger Platz 1, 13353 Berlin, Germany; ^5^Charité-Universitätsmedizin Berlin, Medizinische Klinik mit Schwerpunkt Rheumatologie und Klinische Immunologie, Charité Platz 1, 10117 Berlin, Germany; ^6^Deutsches Rheuma-Forschungszentrum, a Leibniz Institute, Charité Platz 1, 10117 Berlin, Germany; ^7^Dynamic and Functional In Vivo Imaging, Veterinary Medicine, Freie Universität Berlin, Germany; ^8^Charité-Universitätsmedizin Berlin, NeuroCure Clinical Research Center, Charité Platz 1, 10117 Berlin, Germany

## Abstract

In neuroinflammatory and neurodegenerative disorders such as multiple sclerosis, mitochondrial damage caused by oxidative stress is believed to contribute to neuroaxonal damage. Previously, we demonstrated that exposure to hydrogen peroxide (H_2_O_2_) alters mitochondrial morphology and motility in myelinated axons and that these changes initiate at the nodes of Ranvier, where numerous sodium channels are located. Therefore, we suggested that mitochondrial damage may lead to ATP deficit, thereby affecting the efficiency of the sodium-potassium ATPase and eventually leading to sodium overload in axons. The increased intra-axonal sodium may revert the axonal sodium-calcium exchangers and thus may lead to a pathological calcium overload in the axoplasm and mitochondria. Here, we used the explanted murine ventral spinal roots to investigate whether modulation of sodium or calcium influx may prevent mitochondrial alterations in myelinated axons during exogenous application of H_2_O_2_ inducing oxidative stress. For that, tetrodotoxin, an inhibitor of voltage-gated sodium ion channels, and ruthenium 360, an inhibitor of the mitochondrial calcium uniporter, were applied simultaneously with hydrogen peroxide to axons. Mitochondrial shape and motility were analyzed. We showed that inhibition of axonal sodium influx prevented oxidative stress-induced morphological changes (i.e., increase in circularity and area and decrease in length) and preserved mitochondrial membrane potential, which is crucial for ATP production. Blocking mitochondrial calcium uptake prevented decrease in mitochondrial motility and also preserved membrane potential. Our findings indicate that alterations of both mitochondrial morphology and motility in the contexts of oxidative stress can be counterbalanced by modulating intramitochondrial ion concentrations pharmacologically. Moreover, motile mitochondria show preserved membrane potentials, pointing to a close association between mitochondrial motility and functionality.

## 1. Introduction

Multiple sclerosis (MS) is a chronic inflammatory disease of the central nervous system (CNS) that affects approximately 2.5 million people worldwide [1]. The pathological hallmarks of MS include inflammation, demyelination, and neurodegeneration; however, its pathogenesis and the relationship between those three aspects are not completely understood [1].

In this context, mitochondria have emerged as one of the key players that are affected by inflammation and contribute to neuroaxonal loss [2–4]. During neuroinflammatory events in MS, activated CNS-invading leukocytes, as well as microglia, are potential sources of reactive oxygen species (ROS), mainly via increased activation of nicotinamide adenine dinucleotide phosphate (NADPH) oxidases [5–8]. It is assumed that excessive ROS production may lead to oxidative stress and consequently to the inhibition of adenosine triphosphate (ATP) production. Activation of oxidative phosphorylation under pathological stress conditions may also lead to increased chances of electron slippage to oxygen and the formation of additional detrimental ROS [9–11]. In a physiological state, cells have mechanisms to cope with increased ROS production [12, 13]. However, sustained inflammation and oxidative stress may lead to irreversible damage in mitochondria and affect the survivability of the cells [14].

To investigate the impact of oxidative stress on neuroaxonal mitochondria, we have developed an ex vivo model to monitor mitochondrial alterations in murine spinal roots [15, 16]. We focused on ventral spinal roots because they consist predominantly of efferent motor axons and are thicker than dorsal roots making them easier to handle. Using this model, we previously showed that oxidative stress alters both mitochondrial morphology (increases mitochondrial circularity and decreases mitochondrial area and length) and mitochondrial motility (reduces the percentage of moving mitochondria, length of their trajectories and their velocity) [17]. We also observed that, following an oxidative insult, all these alterations consistently initiate at the nodes of Ranvier [17].

In axons, voltage-gated sodium channels (NaV) are mainly located near the nodes of Ranvier [18]. In the presence of oxidative stress, mitochondrial damage may lead to reduced ATP generation [19] and the consequent failure of the sodium-potassium-ATPase (Na^+^/K^+^-ATPase), leading to sodium (Na^+^) accumulation inside the axons [20]. Moreover, in a degeneration paradigm using dorsal root ganglion cells, it has been demonstrated that influx of Na^+^ via NaV contributes to intraneuronal Na^+^ accumulation [13]. To compensate for the excess of intracellular Na^+^ in the presence of a dysfunctional Na^+^/K^+^-ATPase, the axonal sodium-calcium exchanger (NCX) may start acting in a reverse mode, causing axonal calcium (Ca^2+^) overload [5, 13, 20].

High cytosolic Ca^2+^ concentration directly impacts mitochondria, which in turn are part of the Ca^2+^ buffering system of cells [13, 21, 22]. Tightly regulated intracellular Ca^2+^ homeostasis is crucial because an excessive mitochondrial Ca^2+^ uptake may lead to the opening of the permeability transition pore (PTP), resulting in apoptosis [9, 14]. A mitochondrial Ca^2+^ uniporter (MCU) transports Ca^2+^ into the mitochondrial matrix [19]. It has been shown that overexpression of MCU and subsequent mitochondrial Ca^2+^ overload results in neuronal death, both *in vitro* and *in vivo* [23]. Moreover, mitochondria are linked to motor proteins via Miro-1/2, which have Ca^2+^-sensing structures, suggesting that mitochondrial motility is also Ca^2+^-sensitive [18]. Although during physiological state, a slight increase in mitochondrial Ca^2+^ appears to directly stimulate mitochondrial ATP production by activating Ca^2+^-sensitive enzymes of Krebs' Cycle [21], high levels of Ca^2+^ may lead to the suppression of mitochondrial movement [20].

In neuroinflammation, the assumption that alteration of ion concentrations and neuronal damage are connected is supported by the beneficial effects of ion channel blockers reported in experimental autoimmune encephalitis (EAE), where blocking NaV or voltage-gated Ca^2+^ channels attenuates the disease course [13, 24]. Hence, we hypothesized that the abnormal activity of ion channels at the nodes of Ranvier following oxidative stress may cause the observed mitochondrial alterations [5, 13, 24].

Thus, we investigated here if preventing Na^+^ overload within axons and Ca^2+^ overload within mitochondria using the NaV blocker tetrodotoxin (TTX) and the MCU inhibitor ruthenium 360 (Ru360), respectively, would protect both mitochondria and axons from oxidative-stress mediated damage.

## 2. Material and Methods

### 2.1. Ethics Statement

All experimental procedures were approved by the regional animal study committee of Berlin (Landesamt für Gesundheit und Soziales Berlin). Animal experiments were conducted in strict accordance with Directive 2010/63/EU of the European Parliament and of the European Council of 22 September 2010. Female and male mice (8-10 weeks old) were used for the experiments. The mice were housed and maintained in a temperature-controlled environment on a 12 h light-dark cycle.

### 2.2. Preparation and Maintenance of Ventral Spinal Roots

Ventral spinal roots were prepared as described previously [15]. Briefly, C57BL/6 mice were deeply anesthetized with isoflurane before cervical dislocation. After separating the connective tissue, the dorsal side of the spinal cord was exposed, and the vertebrae were cut laterally from rostral to caudal. The spinal cord was sectioned at the thoracic level and the ventral spinal roots were cut distal to the spinal cord. Together with the attached spinal roots, the explanted spinal cord was then placed into artificial cerebrospinal fluid (aCSF), saturated with carbogen (95% O_2_ and 5% CO_2_), and adjusted to a pH of 7.3-7.4. Under a dissecting microscope, the lumbar ventral roots were finally selected and separated from the spinal cord. Explanted ventral roots were maintained in aCSF, containing the following solutions: Solution I – 124 mM NaCl, 1.25 mM NaH_2_PO_4_, 10.0 mM Glucose, 1.8 mM MgSO_4_, 1.6 mM CaCl_2_, 3.00 mM KCl; Solution II – 26.0 mM NaHCO_3_. Both solutions were mixed immediately before use.

### 2.3. Induction of Oxidative Stress and Treatment Groups

All experiments were conducted in a submerged incubation chamber (Brain Slice Keeper-BSK 6 Scientific Systems Design Inc., Ontario, Canada), allowing up to five different treatment conditions and continuous carbogen perfusion of each submersion well throughout the entire process. Although the BSK 6 has 6 individual tubes to supply gas to each of the six wells, one tube had to be used to carbogenate the aCSF stock and therefore only 5 wells were available for the experiments.

To assess the effect of TTX and Ru360 on mitochondrial alterations induced by oxidative stress, we assigned spinal roots randomly to the following experimental groups: a) Negative controls of TTX experiments consisted of axons incubated with aCSF for 30 min at room temperature (RT). Negative controls of Ru360 experiments consisted of axons incubated with the corresponding solvent dimethyl sulfoxide (DMSO) at 1 *μ*l/ml (0,001%) for 30 min at RT. This concentration corresponded to the one used to solve Ru360. DMSO does not exert an effect on investigated mitochondrial parameters (data not shown). We also refer to the negative groups as “untreated groups”. b) In the oxidatively-stressed control group, ventral spinal roots were incubated with 100 *μ*M H_2_O_2_ for 30 min at RT along with the corresponding vehicle (aCSF for TTX experiments, DMSO for Ru360 experiments). We also refer to this group as “positive control”. c) Effects of blocking NaV channels on spinal roots were investigated by incubating the spinal roots with 100 nM or 1 *μ*M TTX along with 100 *μ*M H_2_O_2_. d) Effects of blocking mitochondrial Ca^2+^ influx were determined by incubation with 5, 10, or 20 *μ*M Ru360 along with 100 *μ*M H_2_O_2_.

### 2.4. Labeling of Mitochondria, Microscopy, and Analysis of Mitochondrial Dynamics (Morphology and Motility)

After incubation with the treatments, transected ventral spinal roots were washed and transferred into aCSF containing 100 nM MitoTracker® Orange CMTMRos (Life Technologies, Darmstadt, Germany) dissolved in DMSO for 30 min at RT and then washed again with fresh aCSF.

Microscopy and imaging analysis of the ventral spinal roots were performed as previously described [15]. For microscopy, spinal roots were placed on a glass coverslip and transferred to an imaging chamber containing carbogenated aCSF. A custom-built nylon net was placed on top of the spinal roots to prevent them from moving during image acquisition. For all experiments, an inverted laser-scanning confocal microscope adapted for live-cell imaging was used. Experiments with Na^+^ channel blockade were imaged with an LSM 710 (Carl Zeiss, Jena, Germany). Experiments with Ca^2+^ channel blockade were conducted using a Nikon Scanning Confocal A1Rsi+. MitoTracker® Orange was excited at 561 nm with a diode-pumped solid-state (DPSS) laser. Visualization of mitochondria was performed through a 100x (LSM 710, Carl Zeiss) or 60x (Nikon Scanning Confocal A1Rsi+) oil immersion objective. Regions of interest (ROI) were chosen based on the following criteria: 1) clearly visible node of Ranvier 2) well-labeled mitochondria 3) axon with intact myelin sheath and no signs of membrane disruption in regions adjacent to the selected ROI 4) areas at least 2 mm away from the end of the roots. Scrutinizing the spinal roots from the proximal to the distal end, three separate ROI were chosen. For each ROI, a time-lapse (60-second duration, 2 s/frame) with a resolution of 512x512 pixels was recorded. Exposure time and laser power were reduced to minimize photobleaching and phototoxicity.

The first frame of every time-lapse video was used to assess mitochondrial morphology with an automated analysis tool of the Volocity®6.3 software (Perkin Elmer, Rodgau, Germany). To determine the changes in mitochondrial morphology, the following parameters were analyzed: shape factor (4*π*X [Area/Perimeter^2^]), a measure of circularity ranging from 0 to 1, in which “1” indicates a perfect circle, length (*μ*m) and area (*μ*m^2^) of an individual mitochondrion. To assess motility, mitochondria were tracked manually using Volocity®6.3 software (Perkin Elmer, Rodgau, Germany). Any mitochondrion with a displacement of ≥1 *μ*m was considered “mobile”. For experiments with Ru360, mobile mitochondria were further analyzed for track length (*μ*m), the measure of the real distance traveled by a mitochondrion, and velocity (*μ*m/s).

Under physiological and pathological conditions, mitochondrial populations display high heterogeneity within one cell due to their adaption to different energetic states. Thus, to minimize selection bias, large amounts of mitochondria in different axons of several experiments were analyzed and matched.

### 2.5. Assessment of Mitochondrial Membrane Potential

To determine mitochondrial membrane potential, spinal roots were stained with 20 *μ*g/ml 5,5′,6,6′-tetrachloro-1,1′,3,3′-tetraethylbenzimidazolylcarbocyanine iodide (JC-1; Life Technologies, Darmstadt, Germany) in aCSF at RT for 1 h. JC-1 accumulates in mitochondria with intact membrane potential and negative charge. Sufficient accumulation due to unaltered mitochondrial membrane potential leads to the formation of J aggregates and a shift in emitted fluorescence from green (529 nm) to red (590 nm) [25]. To minimize background noise, roots were washed with fresh aCSF before imaging. JC-1 was excited with dual illumination with argon (514 nm) and DPSS (561 nm) lasers.

Red/green fluorescence ratio of JC-1 stained mitochondria determined at a Nikon Scanning Confocal A1Rsi+microscope was used for the analysis of mitochondrial membrane potential. Results of the red/green fluorescence ratio of individual mitochondrion were normalized to the average red/green fluorescence ratio of the untreated group as established by others [26].

### 2.6. Statistical Analysis

Acquired data were analyzed with Prism 8 Software (GraphPad, CA, USA). All datasets were first subjected to D'Agostino and Pearson omnibus K2 normality test and Shapiro-Wilk normality test for Gaussian distribution. Data fitting the criteria for normal distribution were subsequently analyzed using a one-way ANOVA with Bonferroni's post hoc test. Data following a non-parametric distribution were analyzed using a Kruskal-Wallis test followed by a Dunn's post hoc multiple comparisons test. *p* values ≤ 0.05 were considered significant. The significance of the data was further depicted as ∗ implying *p* ≤ 0.05, ∗∗ implying *p* ≤ 0.01, ∗∗∗ implying *p* ≤ 0.001, and ∗∗∗∗ implying *p* ≤ 0.0001. All data are shown in mean ± SEM.

## 3. Results

### 3.1. Blocking Axonal Na^+^ Influx Prevents Oxidative Stress-Induced Morphological Changes in Mitochondria

**Figure 1:**
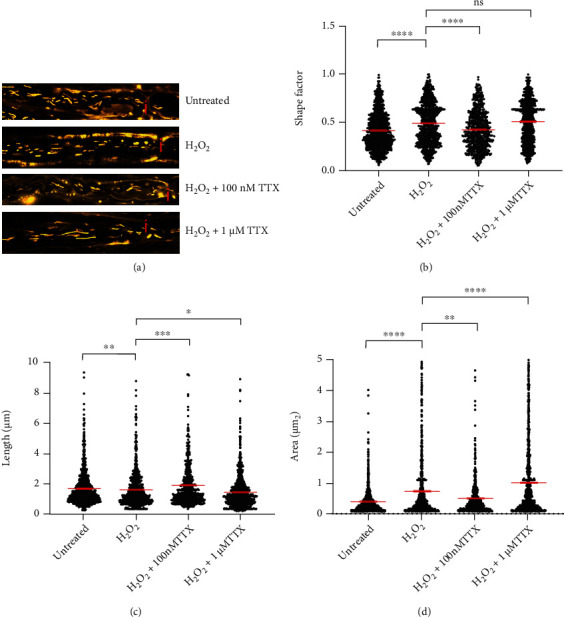
Blocking axonal Na^+^ influx with tetrodotoxin (TTX) prevents oxidative stress-induced mitochondrial morphology alterations. (a) Representative original images of all different experimental conditions; axons incubated with aCSF alone contained elongated mitochondria; incubation with 100 *μ*M H_2_O_2_ led to the generation of smaller and rounder mitochondria, and some diffuse MitoTracker® distribution; axon simultaneously incubated with 100 *μ*M H_2_O_2_ and 100 nM TTX contained elongated mitochondria; axon simultaneously incubated with 100 *μ*M H_2_O_2_ and 1 *μ*M TTX contained short mitochondria but with increased area. (b–d) Shape factor (b), length (c), and area (d) of mitochondria located near the nodes of Ranvier in axons incubated with the above-mentioned treatments. Nodes of Ranvier are marked with a red “i”. ^∗^*p* ≤ 0.05, ^∗∗^*p* ≤ 0.01, ^∗∗∗^*p* ≤ 0.001, and ^∗∗∗∗^*p* ≤ 0.0001. The error bars represent the standard error of mean; *n* = 6 animals and 22 roots; untreated 7 roots, H_2_O_2_ 6 roots, H_2_O_2_+100 nM TTX 4 roots, and H_2_O_2_+1 *μ*M TTX 5 roots.

To investigate the effect of Na^+^ channel blockade on mitochondrial morphology, the explanted ventral spinal roots were treated with 100 *μ*M H_2_O_2_ alone, or 100 *μ*M H_2_O_2_ along with different concentrations of TTX (100 nM or 1 *μ*M). Explants were then imaged using a confocal microscope (Figure 1(a)). Shape factor (Figure 1(b)), mitochondrial length (Figure 1(c)), and mitochondrial area (Figure 1(d)) were analyzed.

During oxidative stress, mitochondrial shape factor (untreated: 0.4148 ± 0.0060; H_2_O_2_: 0.4854 ± 0.0074) and area (untreated: 0.4043 ± 0.0124 *μ*m^2^; H_2_O_2_: 0.7557 ± 0.0335 *μ*m^2^) increased while mitochondrial length decreased (untreated: 1.684 ± 0.0375 *μ*m; H_2_O_2_: 1.5800 ± 0.0431 *μ*m; Figures 1(b) and 1(c)). All observed morphological changes induced by oxidative stress were prevented with 100 nM of TTX (shape factor = 0.4202 ± 0.0079; length = 1.8990 ± 0.0702 *μ*m; area = 0.5247 ± 0.0268 *μ*m^2^; Figures 1(b)–1(d)). In contrast, 1 *μ*M of TTX did not affect the H_2_O_2_-induced increase in shape factor (0.5030 ± 0.0072; Figure 1(b)), but significantly reduced length (1.4400 ± 0.0362 *μ*m, Figure 1(c)) and increased mitochondrial area in comparison to oxidative stress conditions (1.0150 ± 0.0377 *μ*m^2^; Figure 1(d)).

### 3.2. Blocking Axonal Na^+^ Influx Prevents Oxidative Stress-Induced Changes of Mitochondria Motility

**Figure 2:**
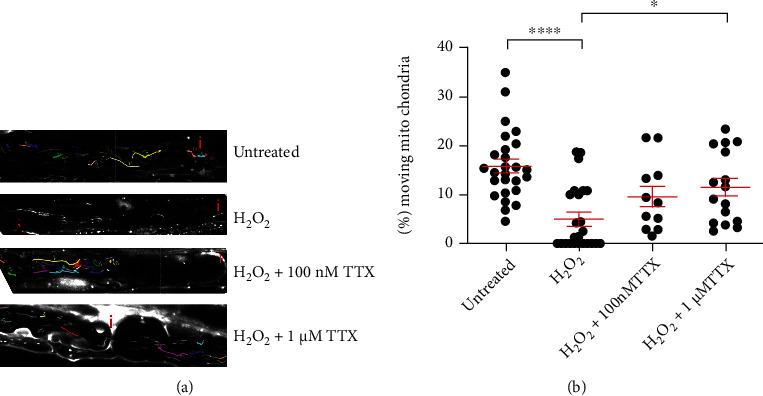
Blocking axonal Na^+^ influx with TTX barely affects mitochondrial motility parameters altered due to oxidative stress. (a) Representative original images of all different experimental conditions: axon incubated with aCSF alone contained multiple moving mitochondria that c5over larger distances in the axon; 100 *μ*M H_2_O_2_ induced a strong reduction of motile mitochondria; axons simultaneously incubated with 100 *μ*M H_2_O_2_ and 100 nM TTX or 1 *μ*M TTX contained more motile mitochondria, covering longer distances. (b) Percentage of moving mitochondria per axon. Nodes of Ranvier are marked with a red “i”. ^∗^*p* ≤ 0.05, ^∗∗∗∗^*p* ≤ 0.0001. The error bars represent the standard error of mean; *n* = 6 animals and 22 roots; untreated 7 roots, H_2_O_2_ 6 roots, H_2_O_2_+100 nM TTX 4 roots, and H_2_O_2_+1 *μ*M TTX 5 roots.

Next, we performed time-lapse imaging and analyzed mitochondrial motility parameters under the above-mentioned experimental conditions (Figure 2(a)). We analyzed the percentage of manually tracked motile mitochondria (Figure 2(b)). The untreated group with aCSF alone showed an average percentage of motile mitochondria of about 16% (15.890 ± 1.395%), while in the presence of 100 *μ*M H_2_O_2_ only around 5% (5.044 ± 1.228%) of mitochondria were motile (Figure 2(b)). Blocking Na^+^ influx with 1 *μ*M TTX prevented the oxidative stress-induced reduction of motile mitochondria (11.460 ± 1.826%; Figure 2(b)). The effect of 100 nM TTX was not significant compared to the H_2_O_2_-treated group (Figure 2(b)).

### 3.3. Blocking Mitochondrial Ca^2+^ Uptake Prevents Oxidative Stress-Induced Alterations of Mitochondrial Length

**Figure 3:**
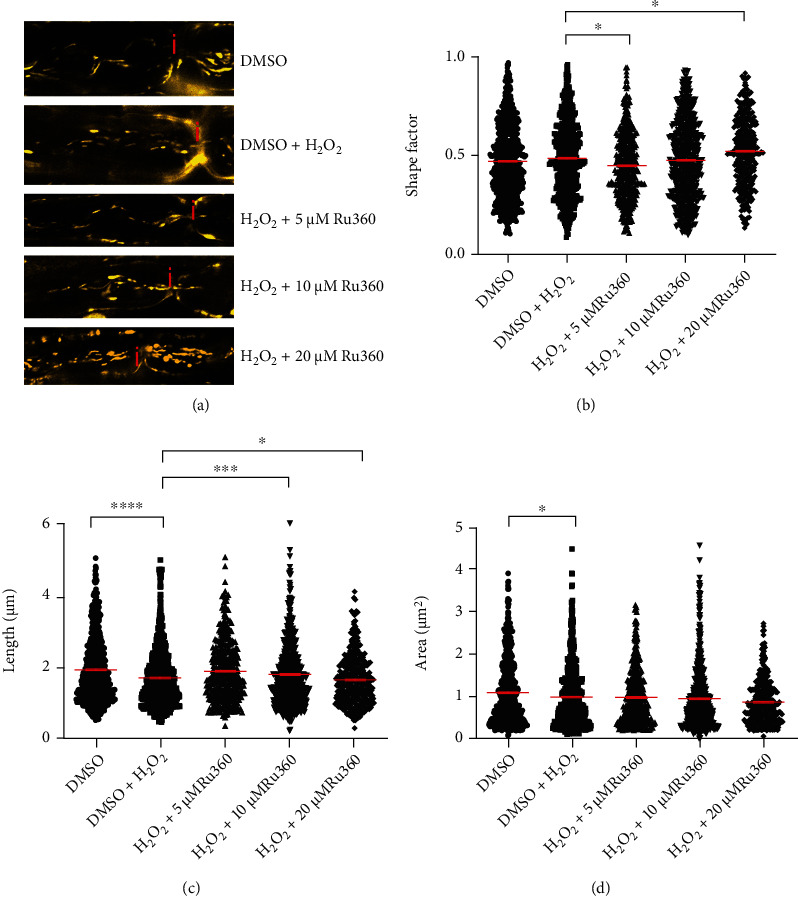
Blocking mitochondrial Ca^2+^ influx with Ru360 alters mitochondrial morphology. (a) Representative original images of all different experimental conditions; axon incubated with DMSO alone showed elongated mitochondria; 100 *μ*M H_2_O_2_ induced shorter and smaller mitochondria; axonal mitochondria exposed to 100 *μ*M H_2_O_2_ and 5 *μ*M Ru360 showed slightly longer and less round morphology than mitochondria exposed to H_2_O_2_ alone; incubation with 100 *μ*M H_2_O_2_ and 10 *μ*M Ru360 led to the formation of longer mitochondria compared with H_2_O2 control; axon incubated with 100 *μ*M H_2_O_2_ and 20 *μ*M Ru360 showed shorter mitochondria than oxidative stress control. (b–d) Mitochondrial shape factor (b), length (c), and area (d) of single mitochondria. Nodes of Ranvier are marked with a red “i”. ^∗^*p* ≤ 0.05, ^∗∗∗^*p* ≤ 0.001, and ^∗∗∗∗^*p* ≤ 0.0001. The error bars represent the standard error of mean; *n* = 7 animals and 29 roots; DMSO 7 roots, H_2_O_2_ 7 roots, H_2_O_2_+5 *μ*M Rui360 4 roots, H_2_O_2_+10 *μ*M Ru360 7 roots, and H_2_O_2_+20 *μ*M Ru360 4 roots.

Then, we examined the influence of mitochondrial Ca^2+^ on mitochondrial morphology. Oxidative stress was induced again with 100 *μ*M H_2_O_2_. Blocking mitochondrial Ca^2+^ influx via mitochondrial Ca^2+^ uniporter channels was performed by simultaneous incubation of mitochondria with H_2_O_2_ and 5, 10, or 20 *μ*M Ru360. We observed that H_2_O_2_ led to a decrease in mitochondrial length (untreated: 1.9260 ± 0.0343 *μ*m; H_2_O_2_: 1.6920 ± 0.0302 *μ*m) and area (untreated: 1.0890 ± 0.0292 *μ*m^2^; H_2_O_2_: 0.9756 ± 0.0268 *μ*m^2^) compared to the untreated group (Figures 3(c) and 3(d)). However, shape factor did not increase under H_2_O_2_-treatment (untreated: 0.4703 ± 0.0072; H_2_O_2_: 0.4841 ± 0.0071) when compared to the untreated group (Figure 3(b)). Blocking mitochondrial Ca^2+^ influx with 5 *μ*M Ru360 prevented changes in shape factor (0.4474 ± 0.0090, Figure 3(b)). A similar trend was observed in roots treated with 10 *μ*M Ru360 (0.4741 ± 0.0083; Figure 3(b)). However, at 20 *μ*M, Ru360 induced an even more pronounced increase in shape factor values (0.5214 ± 0.0095) when compared to the H_2_O_2_-treated group (Figure 3(b)). Incubation with 5 *μ*M Ru360 did not increase mitochondrial length compared to the H_2_O_2_-treated group (1.881 ± 0.0426 *μ*m, Figure 3(c)). In the presence of 10 *μ*M Ru360, mitochondrial length increased (1.7780 ± 0.0390 *μ*m; Figure 3(c)), while at 20 *μ*M Ru360 promoted decrease in mitochondrial length compared to treatment with oxidative stress alone (Figure 3(c)). Regarding area, we did not observe significant alterations in either of the treatment groups (Figure 3(d)).

### 3.4. Blocking Mitochondrial Ca^2+^ Uptake Prevents Reduction of Mitochondrial Motility in Stressed Axons

**Figure 4:**
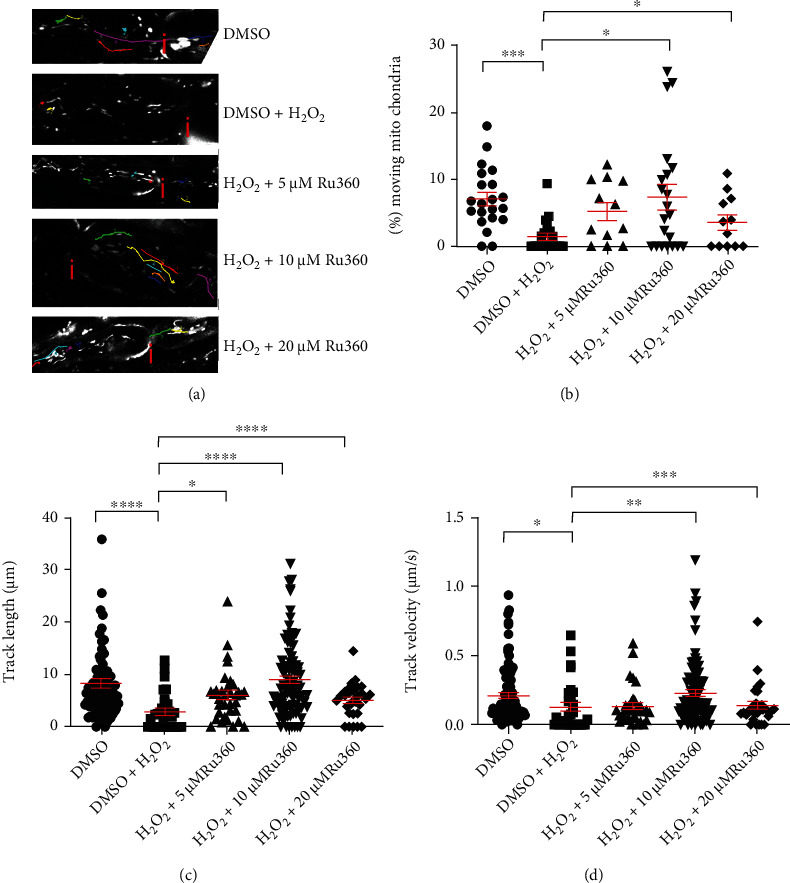
Blocking mitochondrial Ca^2+^ uptake with Ru360 prevents oxidative stress-induced loss of mitochondrial motility. (a) Representative original images of all different experimental conditions: axon incubated with DMSO alone contained multiple moving mitochondria that cover larger distances in the axon.; axon incubated with 100 *μ*M H_2_O_2_, showed few motile mitochondria with short track length; simultaneous incubation with 100 *μ*M H_2_O_2_ and 5 or 20 *μ*M Ru360 led to more motile mitochondria that move longer distances; axon simultaneously incubated with 100 *μ*M H_2_O_2_ and 10 *μ*M Ru360 showed nearly normal mitochondrial motility. (b–d) Quantification of the percentage of motile mitochondria (a), track length (b), and track velocity (c). Nodes of Ranvier are marked with a red “i”. ^∗^*p* ≤ 0.05, ^∗∗^*p* ≤ 0.01, ^∗∗∗^*p* ≤ 0.001, and ^∗∗∗∗^*p* ≤ 0.0001. The error bars represent the standard error of mean; *n* = 7 animals and 29 roots; DMSO 7 roots, H_2_O_2_ 7 roots, H_2_O_2_+5 *μ*M Rui360 4 roots, H_2_O_2_+10 *μ*M Ru360 7 roots, and H_2_O_2_+20 *μ*M Ru360 4 roots.

To investigate the effect of blocking MCU on oxidative stress-induced alterations in mitochondrial motility, we incubated explanted ventral spinal roots with DMSO alone, DMSO plus 100 *μ*M H_2_O_2,_ or with 100 *μ*M H_2_O_2_ along with three different concentrations (5, 10 or 20 *μ*M) of Ru360 (Figure 4(a)).

In the untreated group, we observed an average of 7% (7.103% ± 0.997) of moving mitochondria (Figure 4(b)). H_2_O_2_ at 100 *μ*M caused a significant reduction in motile mitochondria (1.447% ± 0.507) as well as a decrease in track length (untreated: 8.2722 ± 0.8433 *μ*m; H_2_O_2_-treated: 2.8750 ± 0.6442 *μ*m) and track velocity (untreated: 0.2094 ± 0.0210 *μ*m/s; H_2_O_2_-treated: 0.1265 ± 0.0320 *μ*m/s) (Figures 4(a)–4(c)). H_2_O_2_-induced decrease in percentage of motile mitochondria, mitochondrial track length, and track velocity was prevented with 10 *μ*M Ru360 (% of moving mitochondria: 7.393 ± 1.861%; track length: 8.9410 ± 0.7597 *μ*m; track velocity: 0.2293 ± 0.0243 *μ*m/s; Figures 4(a)–4(c)) and 20 *μ*M Ru360 (% of moving mitochondria: 3.549 ± 1.124%; track length: 4.989 ± 0.6025 *μ*m; track velocity: 0.1384 ± 0.0280 *μ*m/s; Figures 4(a)–4(c)). However, in spinal roots treated with 5 *μ*M Ru360, only H_2_O_2_-induced changes for track length (*μ*m, Figure 4(c)) were prevented. No effects were observed on percentage of moving mitochondria or track velocity ((% of moving mitochondria: 5.205 ± 1.325%; track velocity: 0.1331 ± 0.0235 *μ*m/s, Figures 4(a) and 4(d)).

### 3.5. Blocking Axonal Na^+^ Influx Prevents Oxidative Stress-Induced Reduction of Mitochondrial Membrane Potential

**Figure 5:**
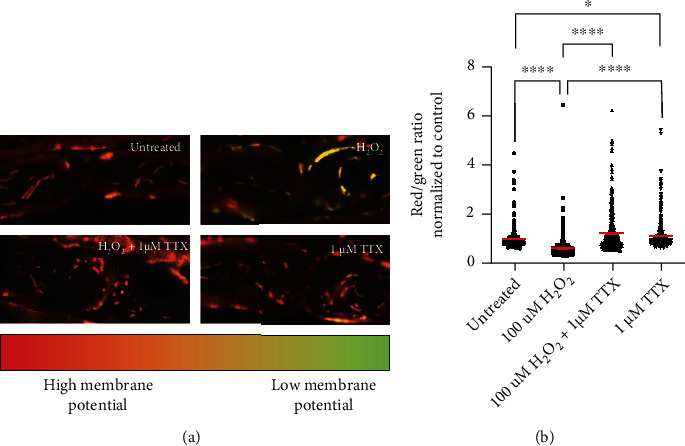
Blocking axonal Na^+^ influx with TTX prevents loss of mitochondrial membrane potential. (a) Representative images of axons in the different treatment groups. The upper left image shows mitochondrial membrane potential in untreated condition. Oxidative stress led to loss of mitochondrial membrane potential (upper right image) and a shift to green fluorescence. TTX prevented the H_2_O_2_ effects (lower left image). The lower right image shows that the application of TTX alone led to preserved mitochondrial membrane potential. (b) Data represent normalized values of individual mitochondria to the mean of the control group (red/green ratio = 1 ± 0.0383). ^∗∗∗∗^*p* ≤ 0.0001. The error bars represent the standard error of mean; *n* = 3 animals and 12 roots; untreated 3 roots, H_2_O_2_ 3 roots, H_2_O_2_+1 *μ*M TTX 3 roots, and 1 *μ*M TTX 3 roots.

Next, we investigated whether inhibition of axonal Na^+^ influx may preserve mitochondrial functionality altered by H_2_O_2_. Four groups of spinal roots were treated for 30 min with either aCSF alone (vehicle control group), 100 *μ*M H_2_O_2_, 100 *μ*M H_2_O_2_+1 *μ*M TTX or 1 *μ*M TTX alone, respectively. Since the incubation chamber permitted the simultaneous assessment of maximally 5 conditions, only the 1 *μ*M TTX concentration, which showed best protecting effects in Figure 2(a), was tested in these experiments. Treated spinal roots were then incubated for 30 min with the ratiometric indicator JC-1. The red/green fluorescence ratio is an indication of the mitochondrial membrane potential and thereby mitochondrial ability to produce ATP (Figure 5(a)).

The application of 100 *μ*M H_2_O_2_ resulted in a shift to green fluorescence (0.6374 ± 0.0291; Figures 5(a) and 5(b)), as a sign of a loss of mitochondrial membrane potential. 1 *μ*M TTX applied simultaneously with 100 *μ*M H_2_O_2_ prevented the loss of mitochondrial membrane potential (untreated: 1.0000 ± 0.0297; 1 *μ*M TTX: 1.2410 ± 0.0432; Figures 5(a) and 5(b)). TTX alone led to higher mitochondrial membrane potential than in the untreated group (TTX: 1.1270 ± 0.0309; Figures 5(a) and 5(b)).

### 3.6. Blocking Mitochondrial Ca^2+^ Uptake Prevents Oxidative Stress-Induced Reduction of Mitochondrial Membrane Potential

**Figure 6:**
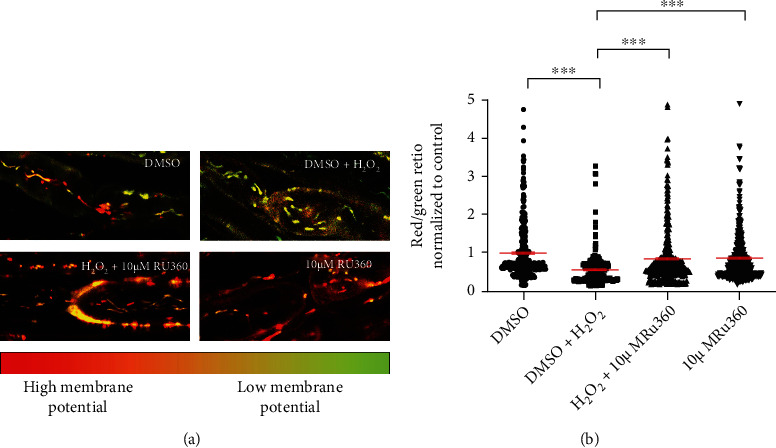
Blocking mitochondrial Ca^2+^ uptake with Ru360 prevents loss of mitochondrial membrane potential. (a) Representative images of axons in the different treatment groups. The upper left image shows mitochondrial membrane potential under negative control conditions containing mitochondria with high (red) and low (green) mitochondrial membrane potential. Oxidative stress led to loss of mitochondrial membrane potential (upper right image) and a shift to green fluorescence. Ru360 prevented the H_2_O_2_ effects (lower left image). The lower right image shows that the application of the Ru360 alone had no effects on mitochondrial functionality compared to control group. (b) Data represent normalized values of individual mitochondria to the mean of the control group (red/green ratio = 1 ± 0.0383). ^∗∗∗^*p* ≤ 0.001. The error bars represent the standard error of mean; *n* = 5 animals and 20 roots; DMSO 5 roots, H_2_O_2_ 5 roots, H_2_O_2_+10 *μ*M Ru360 5 roots, and 10 *μ*M Ru360 5 roots.

To assess effects of Ca^2+^ uptake on mitochondrial functionality, four groups of spinal roots were treated for 30 min with either DMSO (vehicle control group), DMSO +100 *μ*M H_2_O_2_, 100 *μ*M H_2_O_2_+10 *μ*M Ru360 or 10 *μ*M Ru360 alone, respectively. We selected 10 *μ*M Ru360 for these experiments because it was the concentration that showed the best protection against oxidative stress-induced loss of motility (Figure 4(a)). Treated spinal roots were then incubated for 30 min with the ratiometric indicator JC-1. The red/green fluorescence ratio is an indication of the mitochondrial membrane potential and thereby mitochondrial ability to produce ATP (Figure 6(a)).

The application of 100 *μ*M H_2_O_2_ resulted in a shift to green fluorescence (0.5638 ± 0.0250; Figures 6(a) and 6(b)), as a sign of a severe loss of mitochondrial membrane potential. 10 *μ*M Ru360 applied simultaneously with 100 *μ*M H_2_O_2_ prevented the loss of mitochondrial membrane potential and restored it to values close to the untreated group (untreated: 1.0000 ± 0.0383; 10 *μ*M Ru360: 0.8507 ± 0.0395; Figures 6(a) and 6(b)). With Ru360 alone, we did not observe any effects on mitochondrial membrane potential in comparison to DMSO treated condition (Figures 6(a) and 6(b)).

## 4. Discussion

Mitochondrial alterations linked to oxidative stress [9] are reported to occur in the early stages of MS [4, 12] and are believed to contribute to neurodegenerative processes observed in MS patients [2, 27–29]. Therefore, mitochondria have emerged as potential therapeutic targets to limit disease progression [30, 31]. In this study, we investigated using an ex vivo model of peripheral axons [17] whether the effects of oxidative stress on mitochondria can be prevented by targeting pathological ion alterations affecting, in particular, the levels of axonal Na^+^ and mitochondrial Ca^2+^.

In this model, oxidative stress was induced by a 30-minute incubation with 100 *μ*M H_2_O_2_, a concentration that led to reversible structural and functional alterations in mitochondria [32]. We observed oxidative stress-induced decrease in mitochondrial length (Figures 1(c) and 3(c)) as well as a decrease in the number of motile mitochondria (Figures 2(b) and 4(b)). Additionally, consistent with our previous reports [16, 33] and those of others describing inhibition of axonal transport by oxidative stress [34–36], we observed a decrease in both track length and track velocity of mitochondria exposed to 100 *μ*M H_2_O_2_ (Figures 4(c) and 4(d)). The observed reduction in mitochondrial length supports previous findings of our group [17] and may be the consequence of an increase in the fission process, which is induced in stressed and damaged mitochondria to get rid of the damaged portion [37].

We also expected that oxidative stress would damage mitochondria and reduce their functionality in our model causing ATP depletion as it has been reported for highly energy-dependent neuronal cells [9, 38]. We showed a decrease in mitochondrial membrane potential under oxidative stress conditions (Figures 5(a), 5(b), 6(a), and 6(b)). As an intact mitochondrial membrane potential is an important determinant for mitochondrial ATP production via oxidative phosphorylation [39], we assumed ATP depletion in oxidatively injured mitochondria. In a novel CNS model established in our lab, we were indeed able to show decreased ATP levels upon oxidative stress induced by 100 *μ*M H_2_O_2_ [33]. Thus, our paradigm of stressed mitochondria in explanted roots may serve in the future to examine effects of antioxidative interventions on ATP levels.

We previously reported that alterations of mitochondria during oxidative stress initiate at the nodes of Ranvier [17]. NaVs are abundantly present at the nodes of Ranvier and are important for saltatory conduction [40, 41]. In MS lesions, the expression of these channels is reported to be altered [42–44]. In this line, during exposure to H_2_O_2_, blocking NaV with 100 nM TTX prevented the decrease in length and increase in shape factor and area (Figures 1(a)–1(c)). In contrast, 1 *μ*M TTX along with H_2_O_2_ led to the generation of short mitochondria that display however large areas (Figures 1(b) and 1(c)). A large mitochondrial area could reflect either detrimental swelling [45, 46] or fusion [1, 35]. We speculate that in the group treated with H_2_O_2_ and 1 *μ*M TTX, transient mitochondrial fusion followed by fission as reported by Liu et al. [45] may occur. Transient fusion seems to be central for maintaining metabolism and motility [45]. In this line, we observed that 1 *μ*M TTX could prevent the motility decrease and the loss of membrane potential observed in mitochondria exposed to H_2_O_2_ (Figures 2(a), 5(a), and 5(b)).

Interestingly, 1 *μ*M TTX alone induced an elevation of the mitochondrial membrane potential when compared to the untreated group. This may reflect a state defined as mitochondrial hyperpolarization [46, 47]. We hypothesize that the presence of TTX and the consequent reduce Na^+^ influx may lead to a diminished activity of the ATP-dependent Na^+^/K^+^-ATPase and induce an increase of ATP. Thus, in our setup, hyperpolarization may be generated by the ATP-consuming reverse action mode of complex V [46]. The exact mechanism underlying the elevation of the mitochondrial membrane potential with TTX alone will be part of future investigations.

Subsequently to Na^+^ overload, intra-axonal Ca^2+^ accumulation occurs via reverse action mode of NCX, as described in other studies [13, 48]. During axonal Ca^2+^ overload, mitochondria may uptake Ca^2+^ and function as an intracellular Ca^2+^ buffering system [49]. However, excessive intramitochondrial Ca^2+^ may affect mitochondrial function and motility. It has been shown that dynamin-related protein 1 (Drp1), responsible for mitochondrial fission, as well as Miro, connecting mitochondria via other proteins to motor proteins, are directly or indirectly controlled by Ca^2+^ [29, 50–52]. Moreover, mitochondrial swelling seems to be Ca^2+^-related, too [53]. In this case, we demonstrated that inhibition of Ca^2+^ influx into mitochondria with 10 *μ*M Ru360 completely prevents oxidative stress-induced reduction of mitochondrial length and all motility parameters (Figures 3(b) and 4(a)–4(c)). Further, with 10 *μ*M Ru360, we observed preserved mitochondrial membrane potential (Figures 5(a) and 5(b)). Thus, a rise in intramitochondrial Ca^2+^ concentration appears to contribute to mitochondrial alterations during oxidative stress in our model. In the motility experiments, we observed a biphasic effect of Ru360 with similar absolute values for 5 and 20 *μ*M and a clearly different response for 10 *μ*M Ru360. This biphasic effect was observed in all investigated mitochondrial motility parameters, i.e., percentage of motile mitochondria, mitochondrial track length, and track velocity (Figures 3(a)–3(c)).

Our data confirm previous studies that indicated that ion concentrations show no linear correlation with mitochondrial morphology, motility, or membrane potential [54, 55]. While a slight increase in mitochondrial Ca^2+^ concentration may increase mitochondrial ATP production and be beneficial [55], elevated levels of mitochondrial Ca^2+^ may lead to the opening of the PTP with possible detrimental effects [56]. In addition, PTP opening does not only depend on ion concentrations but also on ATP/ADP levels, mitochondrial ROS, fatty acids, and magnesium levels [57–59]. ROS function as signaling molecules, reversibly oxidizing defined structures and thereby regulating transcription or enzyme activity [6, 31, 60–62]. ROS regulates among others the activity of MCU [63], as well as of voltage-gated sodium channels, including NaV1.7 [64]. These potential cellular mechanisms to cope with increased ROS should be kept in mind when dealing with oxidative stress and ion alterations.

Ru360 is a specific inhibitor of the MCU [65, 66]. However, blocking MCU may not result in a complete inhibition of mitochondrial Ca^2+^ influx. As described in metabolically inhibited cells [47], a reverse action mode of mitochondrial Na^+^/Ca^2+^-exchanger may enhance intramitochondrial Ca^2+^ in stressed axons. Additionally, mitochondria closely interact with the endoplasmic reticulum (ER), forming mitochondria-associated membranes (MAMs) [67]. MAMs play a role in the exchange of Ca^2+^ or metabolites [68, 69], mitochondrial fusion and fission processes, and induction of apoptosis [70].

Mitochondria possess different mechanisms of Ca^2+^ influx [71], but also of Ca^2+^ efflux. The two most important mechanisms are via mitochondrial Na^+^/Ca^2+^-exchanger and via 2H^+^/Ca^2+^-exchanger [71, 72]. Mitochondrial Ca^2+^ uptake is therefore most likely directly influenced by intra-axonal Na^+^ concentration because this affects mitochondrial Ca^2+^ efflux mechanisms via mitochondrial Na^+^/Ca^2+^-exchanger. Interestingly, a reverse action mode is also described for mitochondrial Na^+^/Ca^2+^-exchanger in metabolically inhibited cells [48]. Thus, blocking either axonal Na^+^ influx or mitochondrial Ca^2+^ uptake may likely indirectly interfere with other pathways, for example via mitochondria-associated membranes (MAMs) or mitochondrial Na^+^/Ca^2+^-exchanger of a tightly regulated and interconnected Na^+^- Ca^2+^-homeostasis.

## 5. Limitations of the Study

One technical limitation of our setup was the restricted number of experimental conditions that could be conducted simultaneously within one experiment. The size of the incubation chamber and the narrow time-window, in which transplants could be imaged ex vivo, permitted only the comparison of maximally five different culture conditions. Therefore, using this setup, we were unable to compare effects on mitochondria of different concentrations of inhibitors both in the absence and the presence of the oxidative insult.

Therefore, using this setup, we were able to show only effects on mitochondria of different concentrations of inhibitors in the oxidative stress paradigm and not in the absence of H_2_O_2_.

Moreover, although our data indicate that modulation of Ca^2+^ influx with Ru360 protects mitochondria from oxidative stress-induced damage, we could not define which Ca^2+^ concentrations are protective and which concentrations are detrimental for mitochondria. Basically, we attested that the explanted root model was not suitable for intra-axonal Ca^2+^quantification using, for instance, Ca^2+^-sensitive dyes or roots from Ca^2+^ reporter mice.

## 6. Conclusion

In conclusion, explanted murine spinal roots appear to be a suitable model to investigate oxidative stress-induced ion alterations affecting axonal mitochondria, in particular, Na^+^ and Ca^2+^ overload. Using the model, we demonstrated that inhibition of axonal Na^+^ influx prevented oxidative stress-induced alterations of mitochondrial morphology. On the other hand, blocking mitochondrial Ca^2+^ uptake prevented the oxidative stress-induced reduction of both mitochondrial motility and mitochondrial membrane potential, which is crucial for ATP production.

The fact that H_2_O_2_-induced alterations in mitochondria morphology and motility were prevented by pharmacologic inhibitors of NaV and MCU indicates a direct participation of Na^+^ and Ca^2+^ on oxidative stress-mediated mitochondrial changes. Further investigations in this direction are needed to explore the therapeutic potential of the modulation of Na^+^ and Ca^2+^ ion channel for mitochondrial protection during oxidative stress.

## Data Availability

The main data supporting the findings of this study are listed in Tables 1–6 of the Supplementary Materials.

## Supplementary Materials

Supplementary 1 Table 1: summary of morphology parameters of untreated mitochondria, mitochondria under H_2_O_2_ treatment alone, and mitochondria treated with H_2_O_2_ in the presence of 100 nM and 1 *μ*M TTX.

Supplementary 2 Table 2: summary of motility parameters of untreated mitochondria, mitochondria under H_2_O_2_ treatment alone, and mitochondria treated with H_2_O_2_ in the presence of 100 nM and 1 *μ*M TTX.

Supplementary 3 Table 3: summary of morphology parameters of untreated mitochondria, mitochondria under H_2_O_2_ treatment alone, and mitochondria treated with H_2_O_2_ in the presence of 5 *μ*M, 10 *μ*M, and 20 *μ*M Ru360.

Supplementary 4 Table 4: summary of motility parameters of untreated mitochondria, mitochondria under H_2_O_2_ treatment alone, and mitochondria treated with H_2_O_2_ in the presence of 5 *μ*M, 10 *μ*M, and 20 *μ*M Ru360.

Supplementary 5 Table 5: summary of the red-green ratio normalized to untreated mitochondria, mitochondria treated with 100 *μ*M H_2_O_2_ alone or in the presence of 1 *μ*M TTX, and mitochondria treated with 1 *μ*M TTX alone.

Supplementary 6 Table 6: summary of the red-green ratio normalized to untreated mitochondria, mitochondria treated with 100 *μ*M H_2_O_2_ alone or in the presence of 10 *μ*M Ru360, and mitochondria treated with 10 *μ*M Ru360 alone.
