# Supplementary material for: Preventing Axonal Sodium Overload or Mitochondrial Calcium Uptake Protects Axonal Mitochondria from Oxidative Stress-Induced Alterations
Source: Oxid Med Cell Longev. 2022 May 24;2022:6125711. doi: 10.1155/2022/6125711 (PMC9157283; doi:10.1155/2022/6125711)
Supplement: Supplementary 1 — Table 1: summary of morphology parameters of untreated mitochondria, mitochondria under H2O2 treatment alone, and mitochondria treated with H2O2 in the presence of 100 nM and 1 μM TTX. [file 6125711.f1.docx]

|  | **Number of spinal roots** | **Number of analyzed individual objects** | **Shape Factor** | **Length (µm)** | **Area (µm^2^)** |
| --- | --- | --- | --- | --- | --- |
| **Untreated** | 7 | 1010 | 0.4148 ± 0.0060 | 1.684 ± 0.0375 | 0.4043 ± 0.0124 |
| **H_2_O_2_-treated** | 6 | 812 | 0.4854 ± 0.0074 | 1.58 ± 0.0431 | 0.7557 ± 0.0335 |
| **H_2_O_2_ +TTX (100 nM)** | 4 | 622 | 0.4202 ± 0.0079 | 1.899 ± 0.0702 | 0.5247 ± 0.0268 |
| **H_2_O_2_ + TTX (1 µM)** | 5 | 911 | 0.503 ± 0.0072 | 1.44 ± 0.0362 | 1.015 ± 0.0377 |

Table 1: Summary of morphology parameters of untreated mitochondria, mitochondria under H_2_O_2_ treatment alone, and mitochondria treated with H_2_O_2_ in presence of 100 nM and 1 µM TTX. Values are shown as Mean ± SEM.
