# Supplementary material for: Preventing Axonal Sodium Overload or Mitochondrial Calcium Uptake Protects Axonal Mitochondria from Oxidative Stress-Induced Alterations
Source: Oxid Med Cell Longev. 2022 May 24;2022:6125711. doi: 10.1155/2022/6125711 (PMC9157283; doi:10.1155/2022/6125711)
Supplement: Supplementary 2 — Table 2: summary of motility parameters of untreated mitochondria, mitochondria under H2O2 treatment alone, and mitochondria treated with H2O2 in the presence of 100 nM and 1 μM TTX. [file 6125711.f2.docx]

|  | **Number of spinal roots** | **Number of analyzed individual objects** | **Percentage of Motile Mitochondria (%)** |
| --- | --- | --- | --- |
| **Untreated** | 7 | 30 | 15.89 ± 1.395 |
| **H_2_O_2_-treated** | 6 | 11 | 5.044 ± 1.228 |
| **H_2_O_2_ +TTX (100 nM)** | 4 | 14 | 9.670 ± 2.17 |
| **H_2_O_2_ + TTX (1 µM)** | 5 | 16 | 11.46 ± 1.826 |

Table 2: Summary of motility parameters of untreated mitochondria, mitochondria under H_2_O_2_ treatment alone, and mitochondria treated with H_2_O_2_ in presence of 100 nM and 1 µM TTX. Values are shown as Mean ± SEM.
