# Supplementary material for: Preventing Axonal Sodium Overload or Mitochondrial Calcium Uptake Protects Axonal Mitochondria from Oxidative Stress-Induced Alterations
Source: Oxid Med Cell Longev. 2022 May 24;2022:6125711. doi: 10.1155/2022/6125711 (PMC9157283; doi:10.1155/2022/6125711)
Supplement: Supplementary 3 — Table 3: summary of morphology parameters of untreated mitochondria, mitochondria under H2O2 treatment alone, and mitochondria treated with H2O2 in the presence of 5 μM, 10 μM, and 20 μM Ru360. [file 6125711.f3.docx]

|  | **Number of spinal roots** | **Number of analyzed individual objects** | **Shape Factor** | **Length (µm)** | **Area (µm^2^)** |
| --- | --- | --- | --- | --- | --- |
| **Untreated** | 7 | 686 | 0.4703 ± 0.0072 | 1.926 ± 0.0343 | 1.0890 ± 0.0292 |
| **H_2_O_2_-treated** | 7 | 687 | 0.4841 ± 0.0071 | 1.692 ± 0.0302 | 0.9756 ± 0.0268 |
| **H_2_O_2_ +Ru360 (5 µM)** | 4 | 378 | 0.4474 ± 0.0090 | 1.881 ± 0.0426 | 0.9706 ± 0.0335 |
| **H_2_O_2_ + Ru360 (10 µM)** | 7 | 576 | 0.4741 ± 0.0083 | 1.778 ± 0.0390 | 0.9494 ± 0.0307 |
| **H_2_O_2_ + Ru360 (20 µM)** | 4 | 345 | 0.5214 ± 0.0095 | 1.648 ± 0.0376 | 0.8670 ± 0.02806 |

Table 3: Summary of morphology parameters of untreated mitochondria, mitochondria under H_2_O_2_ treatment alone, and mitochondria treated with H_2_O_2_ in presence of 5 µM, 10 µM and 20 µM Ru360. Values are shown as Mean ± SEM.
