# Supplementary material for: Preventing Axonal Sodium Overload or Mitochondrial Calcium Uptake Protects Axonal Mitochondria from Oxidative Stress-Induced Alterations
Source: Oxid Med Cell Longev. 2022 May 24;2022:6125711. doi: 10.1155/2022/6125711 (PMC9157283; doi:10.1155/2022/6125711)
Supplement: Supplementary 4 — Table 4: summary of motility parameters of untreated mitochondria, mitochondria under H2O2 treatment alone, and mitochondria treated with H2O2 in the presence of 5 μM, 10 μM, and 20 μM Ru360. [file 6125711.f4.docx]

|  | **Number of spinal roots** | **Number of analyzed individual objects** | **Percentage of Motile Mitochondria (%)** | **Track Length (µm)** | **Track Velocity (µm/s)** |
| --- | --- | --- | --- | --- | --- |
| **Untreated** | 7 | 94 | 7.103 ± 0.9968 | 8.272 ± 0.8433 | 0.2094 ± 0.0210 |
| **H_2_O_2_-treated** | 7 | 30 | 1.447 ± 0.5074 | 2.875 ± 0.6442 | 0.1265 ± 0.0320 |
| **H_2_O_2_ +Ru360 (5 µM)** | 4 | 34 | 5.205 ± 1.325 | 5.903 ± 0.8408 | 0.1331 ± 0.0235 |
| **H_2_O_2_ + Ru360 (10 µM)** | 7 | 88 | 7.393 ± 1.861 | 8.941 ± 0.7597 | 0.2293 ± 0.0243 |
| **H_2_O_2_ + Ru360 (20 µM)** | 4 | 29 | 3.549 ± 1.124 | 4.989 ± 0.6025 | 0.1384 ± 0.0280 |

Table 4: Summary of motility parameters of untreated mitochondria, mitochondria under H_2_O_2_ treatment alone, and mitochondria treated with H_2_O_2_ in presence of 5 µM, 10 µM and 20 µM Ru360. Values are shown as Mean ± SEM.
