# Supplementary material for: Preventing Axonal Sodium Overload or Mitochondrial Calcium Uptake Protects Axonal Mitochondria from Oxidative Stress-Induced Alterations
Source: Oxid Med Cell Longev. 2022 May 24;2022:6125711. doi: 10.1155/2022/6125711 (PMC9157283; doi:10.1155/2022/6125711)
Supplement: Supplementary 5 — Table 5: summary of the red-green ratio normalized to untreated mitochondria, mitochondria treated with 100 μM H2O2 alone or in the presence of 1 μM TTX, and mitochondria treated with 1 μM TTX alone. [file 6125711.f5.docx]

|  | **Number of spinal roots** | **Number of analyzed individual objects** | **Red-green ratio normalized to** |
| --- | --- | --- | --- |
| **Untreated** | 3 | 259 | 1.000 ± 0.0297 |
| **H_2_O_2_-treated** | 3 | 258 | 0.6374 ± 0.0291 |
| **H_2_O_2_ +TTX (1 µM)** | 3 | 356 | 1.241 ± 0.0432 |
| **TTX (1 µM)** | 3 | 375 | 1.127 ± 0.0309 |

Table 5: Summary red-green ratio normalized to untreated mitochondria, mitochondria treated with 100 µM H_2_O_2_ alone or in presence of 1 µM TTX and mitochondria treated with 1 µM TTX alone. Values are shown as Mean ± SEM.
