# Supplementary material for: Preventing Axonal Sodium Overload or Mitochondrial Calcium Uptake Protects Axonal Mitochondria from Oxidative Stress-Induced Alterations
Source: Oxid Med Cell Longev. 2022 May 24;2022:6125711. doi: 10.1155/2022/6125711 (PMC9157283; doi:10.1155/2022/6125711)
Supplement: Supplementary 6 — Table 6: summary of the red-green ratio normalized to untreated mitochondria, mitochondria treated with 100 μM H2O2 alone or in the presence of 10 μM Ru360, and mitochondria treated with 10 μM Ru360 alone. [file 6125711.f6.docx]

|  | **Number of spinal roots** | **Number of analyzed individual objects** | **Red-green ratio normalized to** |
| --- | --- | --- | --- |
| **Untreated** | 5 | 442 | 1.000 ± 0.0383 |
| **H_2_O_2_-treated** | 5 | 341 | 0.5638 ± 0.0250 |
| **H_2_O_2_ +Ru360 (10 µM)** | 5 | 389 | 0.8507 ± 0.0395 |
| **Ru360 (10 µM)** | 5 | 505 | 0.8708 ± 0.0389 |

Table 6: Summary red-green ratio normalized to untreated mitochondria, mitochondria treated with 100 µM H_2_O_2_ alone or in presence of 10 µM Ru360 and mitochondria treated with 10 µM Ru360 alone. Values are shown as Mean ± SEM.
